# Beauty is in the efficient coding of the beholder

**DOI:** 10.1098/rsos.160027

**Published:** 2016-03-02

**Authors:** Julien P. Renoult, Jeanne Bovet, Michel Raymond

**Affiliations:** 1 Institute for Arts, Creations, Theories and Esthetics, UMR8218 CNRS-University of Paris 1, Paris, France; 2 Institute for Advanced Study in Toulouse, Toulouse, France; 3 Institute for Evolutionary Sciences, UMR 5554 CNRS-University of Montpellier, Montpellier, France

**Keywords:** sexual selection, sparse coding, sensory bias, sensory exploitation, face, aesthetics

## Abstract

Sexual ornaments are often assumed to be indicators of mate quality. Yet it remains poorly known how certain ornaments are chosen before any coevolutionary race makes them indicative. Perceptual biases have been proposed to play this role, but known biases are mostly restricted to a specific taxon, which precludes evaluating their general importance in sexual selection. Here we identify a potentially universal perceptual bias in mate choice. We used an algorithm that models the sparseness of the activity of simple cells in the primary visual cortex (or V1) of humans when coding images of female faces. Sparseness was found positively correlated with attractiveness as rated by men and explained up to 17% of variance in attractiveness. Because V1 is adapted to process signals from natural scenes, in general, not faces specifically, our results indicate that attractiveness for female faces is influenced by a visual bias. Sparseness and more generally efficient neural coding are ubiquitous, occurring in various animals and sensory modalities, suggesting that the influence of efficient coding on mate choice can be widespread in animals.

## 1. Introduction

Darwin thought of mate choice as a pure aesthetic experience, a selection and celebration of beauty for its own sake [1–3]. His view has not been embraced by modern evolutionary biology, for which choice evolves because ornaments indicate the quality of their owners [4]. Yet little is known about the origin of the association between ornaments and choice, that is, the primary step needed for any further coevolutionary process to run. Could the original association be free of any utilitarian strings and thus match the Darwinian, aesthetic view of mate choice? Does the initiating mechanism continue to influence mate choice in conjunction with other mechanisms?

Perceptual biases, which encompasses both sensory and cognitive biases, are frequently proposed to initiate the choice-ornament coevolution [5]. The mechanism assumes that choices arise as by-products of the adaptation of perceptual systems to tasks unrelated to sexual selection [6]. All perceptual systems evolve biases in response to selection by the environment, and mating biases are therefore inevitable [7]. Nevertheless, the importance of perceptual biases in mate choice is rarely assessed because biases are mostly unknown or, when known, are restricted to a specific taxon (e.g. [5,6]). A notable exception is the preference for symmetry, which seems to occur in a wide range of taxa and which has been proposed to have a perceptual bias origin [8]. In this study, we identify a different perceptual bias that influences attraction to mates; a bias that is potentially universal, occurring with any stimulus processed by any sensory system: the efficient coding bias.

There is ample evidence that perceptual systems are adapted to efficiently code information from the natural environments, that is, the type of environment where our ancestors lived [9,10]. Efficient coding is achieved notably by removing redundant signals from stimuli [11]. In an image, redundancy occurs when the value at a given pixel can be partly predicted by the values at neighbouring pixels. In primates, this type of redundancy is processed by retinal ganglion cells and by the lateral geniculate nucleus [12]. Another important source of redundancy occurs in the so-called fourth-order structure of an image and is captured by analysing sparseness in feature coding. An image feature, for example, a line with a specific orientation, is sparsely coded if a relatively small number of encoders (e.g. neurons) are active at the same time. In primates, the fourth-order structure of visual stimuli is essentially processed by the simple cells of the primary visual cortex (V1) [10].

The efficient coding strategy is adaptive in at least two ways. With redundancies discarded, signals are compacted and are thus more rapidly and precisely processed, which facilitates memory storing and retrieving [13]. In addition, vision is remarkably costly: in humans, information coding and processing within the visual system alone accounts for 2.5–3.5% of a resting body’s overall energy needs [14]. Because it requires a limited number of active neurons, sparse coding therefore allows saving metabolic resources [10,15].

A stimulus that incidentally exhibits the same spatial structures than that of natural environments provides the observer with direct benefits because it is most efficiently coded by the sensory system. We predict that observers have evolved a preference bias for such stimuli. In this article, we tested this prediction by studying how the degree of similarity in fourth-order spatial structures between natural scenes (forest and open landscapes) and females’ face correlates with the attractiveness of these faces for men.

## 2. Material and methods

### 2.1 Image datasets

Caucasian women aged between 18 and 26 were recruited by social network and advertising in different cities from France, between 2010 and 2011. The face of each woman was photographed using a Canon EOS 20D camera and a 50 mm lens with a standardized procedure (lens-face distance set to 1 m, controlled lighting conditions, fixed camera settings).

All photographs were post-processed using Adobe Photoshop to normalize size (photographs were aligned on eye position, with a fixed distance between eyes and chin). We analysed two sets of images that have been collected for the purpose of other studies on womens attractiveness (for dataset 1, see [16]; for dataset 2, see [17]). The two datasets represent faces from different women and differ in how images have been further post-processed (see the electronic supplementary material, figure S1). In dataset 1 (*n*=166), the background was replaced by a uniform black colour, hair and necks were blurred and images were converted to greyscale using the rgb2gray function in Matlab. In dataset 2 (*n*=68), the background was replaced by a uniform neutral grey; neck and shoulders were removed. Photographs were stored in .jpg format.

### 2.2 Scoring attractiveness

The attractiveness of woman faces was evaluated by Caucasian men recruited on public places in Montpellier, France. In a first study (dataset 1), a Delphi-based computer program was constructed to randomly display one face at a time to 169 men (mean: 36 years). The photographs (faces’ height on the screen=490 pixels for dataset 1 and 460 pixels for dataset 2) were presented on a 13 inch screen at a 1366×768 resolution. The observers were seated in a chair, facing the screen at a distance of 50 cm. For each face, the rater was instructed to move a cursor between 0 (lowest attractiveness) and 20 (highest attractiveness). The program stored a value between 0 and 100 by linearly scaling rater’s score. Each rater assessed attractiveness of 30 different faces. In a second study (dataset 2), another computer program was constructed to randomly display pairs of images to 156 men (mean: 36 years). For each pair, the rater was instructed to click on the photograph of the face he found the most attractive. The position of the photograph on the screen (left or right) was ascribed randomly. Each rater assessed 30 distinct pairs corresponding to 60 faces, which could therefore be sorted for attractiveness. A score of attractiveness was then calculated as the average rank.

For both studies, if the rater knew one of the women he had to evaluate, the trial was removed. Also, the first photograph/pair of photographs seen by each participant was not used for the analyses, because the task could require some habituation. Three photographs/pair of photographs, randomly chosen from those previously assessed were displayed again at the end of the trial to test for reliability of judgement. If both assessments differed (i.e. more than 10% differences for dataset 1 or incongruent click for dataset 2) more than once, the rater was qualified unreliable and his answers removed from the analyses. Finally, we used ratings from 119 and 142 men in dataset 1 and dataset 2, respectively.

### 2.3 Sparse coding

We first whitened all images in order to model signal processing before the visual cortex, and to facilitate convergence of the sparse coding algorithm. We used the whitening procedure described in [18]. It is based on a circularly symmetric low-pass filter, which attenuates low frequencies and boosts high frequencies (except the very highest frequencies). The frequency response of the filter is

2.1 $$R(\;f)=f{\text{e}}^{-{\left(\;f/f_{o}\right)}^{4}},$$

with a cut-off of highest frequencies, *f*_o_, of 200 cycles/picture. Such a filter roughly resembles the spatial frequency response of retinal ganglion cells [18]. In addition, it decorrelates first- and second-order statistics of the image, leaving the higher-order redundancies that are analysed through sparse coding.

**Figure 1.**
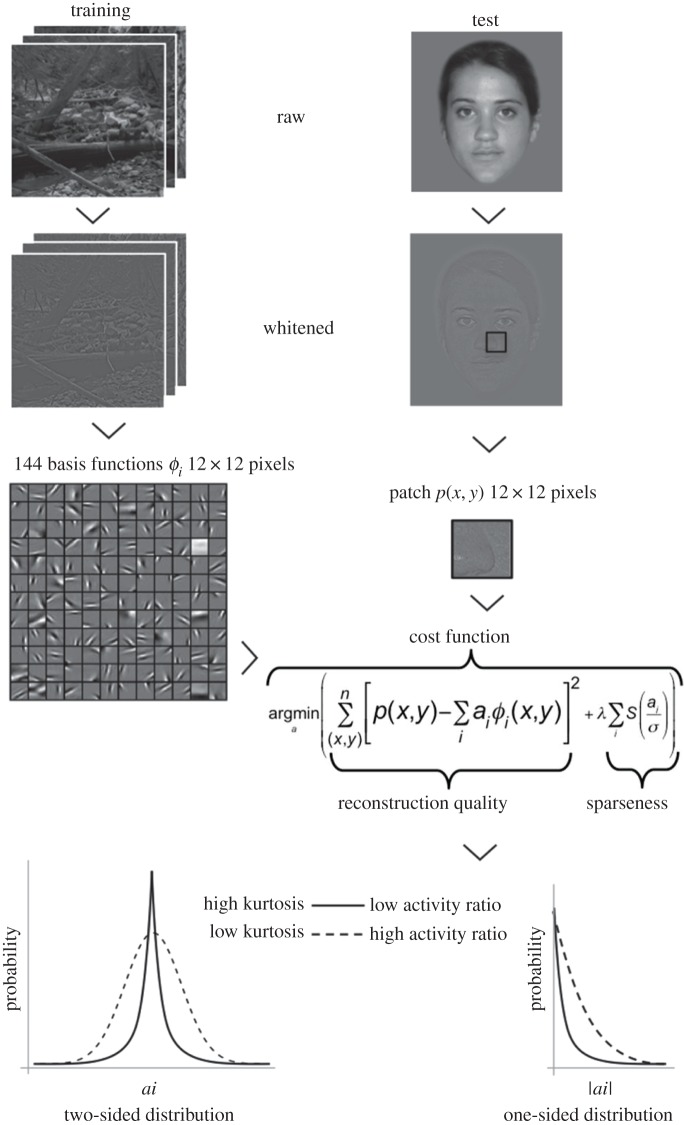
Workflow for estimating the sparseness of face images. We first trained 144 basis functions *Φ*_*i*_ (of size 12×12) to reproduce natural scenes using the same *sparseness* algorithm and model parameters as in [9]. Then, for each face image we convolved basis functions with *n* patches *p*(*x*,*y*) and worked out the combination of activity coefficients *a*_*i*_ (i.e. the weights of *Φ*_*i*_) that minimizes a cost function. Coordinates (*x*,*y*) of patch centres take all possible values along the width and the height of an image, respectively. The cost function accounts for both the quality of face reconstruction and the sparseness of *a*_*i*_ distribution. *λ* determines the relative importance of these two components. The quality of reconstruction is given by the error mean square. During minimization, sparseness of *a*_*i*_ was estimated by summing each coefficient activity (scaled by constant *σ*) passed through a nonlinear function *S* (see [8]). Here, *λ*=0.044 and *σ*=0.316. Analyses were repeated with 64 basis functions (8×8), and with 256 basis functions (16×16). We calculated two measures of sparseness for each face: the kurtosis of *a*_*i*_ distribution and the activity ratio (using |*ai*|). A sparse representation of face has high kurtosis and low activity ratio.

To study the sparseness of faces, we first trained an artificial neural network to reconstruct whitened images of natural scenes with a sparse coding algorithm, a step known as dictionary learning in visual computing (figure 1). As in a principal component analysis (PCA), the algorithm learns a set of basis functions with the goal to reconstruct any patch of an image from a linear combination of basis functions. Contrary to the PCA, however, the sparseness algorithm does not constrain basis functions to be orthogonal to each other. Rather, it maximizes the sparseness of the density function of weights, that is, for a given patch the weight associated to each basis function should be zero in most instances (figure 1). We used the same algorithm, model parameters and training images (*n*=10) as in [9], who showed that the basis functions trained this way describe detectors of light changes (i.e. luminance contrasts) with sensitivity properties similar to that of the simple cells located in the primary visual cortex of primates (V1 area). We trained three dictionaries, with the size *h*^2^ of basis functions set to 8×8, 12×12 or 16×16 pixels. Varying the size of basis functions is equivalent to modelling different sizes for the receptive field of V1 neurons. In each dictionary, the number of basis functions was set to *h*^2^.

Then, for each face representation we extracted patches of size *h*^2^, centred on every pixel. Accounting for the effect of edges, this represents *n*=316 999, 312 471 and 307 975 patches for an image of size 500×650, with *h*=8, 12 and 16, respectively. We removed patches representing the background only (i.e. all black patches in dataset 1 and all neutral grey patches in dataset 2) to avoid overestimating sparseness in pictures with a high amount of background. We used the conjugate gradient descent algorithm implemented in the *sparsenet* package for Matlab [9] to look for the coefficients *a*_*i*_ of the linear combination of basis functions that minimizes the cost function described in figure 1, which aims at reconstructing each image patch from the dictionary while maximizing both the precision of patch reconstruction and the kurtosis of *a*_*i*_. The sparseness of each face representation was then estimated with two different measures, the mean kurtosis of *a*_*i*_:

2.2 $$\text{kurtosis}=\frac{1}{n}\sum_{1}^{n}\frac{1}{h^{2}}\sum_{i=1}^{h^{2}}\frac{\left(a_{i}-\bar{a}\right)}{\sigma {\left(a\right)}^{4}}$$

and the mean activity ratio [19] adapted to ‘population sparseness’ [20]:

2.3 $$\text{activity ratio}=\frac{1}{n}\sum_{1}^{n}\frac{{\left((1/h^{2})\sum_{i=1}^{h^{2}}\left|a_{i}\right|\right)}^{2}}{(1/h^{2})\sum_{i=1}^{h^{2}}a_{i}^{2}}.$$

### 2.4 Facial symmetry and skin roughness

For each face, we further estimated its symmetry using a classical method based on landmark points [21,22]; for details, see the electronic supplementary material, figure S2). We also analysed the roughness of skin texture by calculating entropy (function *entropyfilt* in Matlab), a measure of randomness in pixel distribution. A unique roughness value was attributed to each face by averaging entropies calculated for every 12×12 squares embedded within three 180×100 rectangles; one on each cheek and one on the forehead (electronic supplementary material, figure S2).

### 2.5 Statistical analyses

We analysed the two datasets separately. Using the statistical software R, we performed linear models with *attractiveness* as a response variable, facial *symmetry*, skin *roughness*, *age* of women and sparseness (either *kurtosis* or *activity ratio*) as explanatory variables. The significance of each term was assessed from the full model including all four explanatory variables. Model assumptions were validated graphically by plotting the residuals versus fitted values to evaluate homogeneity, the residuals versus each explanatory variable to evaluate independence (in multivariate models only), and by drawing a QQ-plot of standardized residuals to assess normality.

## 3. Results and discussion

### 3.1 Sparseness and attractiveness are correlated

**Figure 2.**
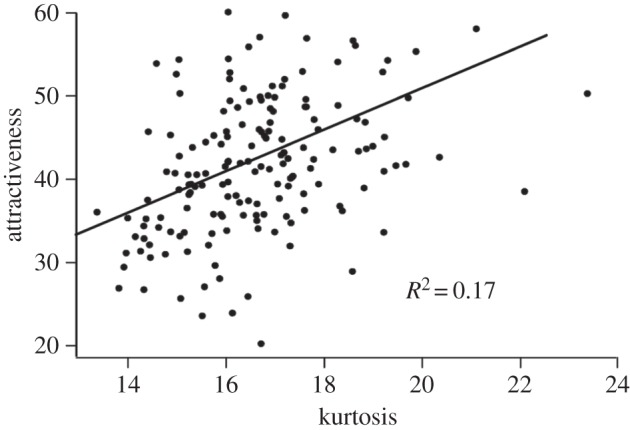
Correlation between attractiveness and kurtosis of *a*_*i*_ distribution in dataset 1. Attractiveness is an average score within the interval [0; 100]. The number of basis functions and the size of receptive fields were set to 16×16.

With dataset 1, variation in sparseness significantly explained variation in attractiveness independently of the measure of sparseness and the size of receptive fields (table 1; figure 2). With dataset 2, sparseness was significantly or marginally significant except in one case (activity ratio with 8×8 basis functions, table 1). The Spearman coefficient of determination (*R*^2^) between attractiveness and sparseness varied between 0.17 (dataset 1; electronic supplementary material, table S1) and 0.04 (dataset 2).

**Table 1.** Summary of the regression models. (The models tested were attractiveness ∼*β*_0_+*β*_1_×sparseness+*β*_2_×symmetry+*β*_3_×roughness+*β*_4_×age+*ε*; *ε*∼*N*(0,*σ*^2^). Results are provided in the form *β*_*i*_(*P*(*t*)), with *P*(*t*) giving the significance of the test *β*_*i*_=0. *β*_2_ and *β*_4_ were never significantly different from 0 (electronic supplementary material, table S1). Sparseness is measured either as kurtosis or as activity ratio. Result for skin roughness is given for the model with kurtosis as a measure of sparseness only.)

	dataset 1			dataset 2		
size of receptive fields	kurtosis	activity ratio	roughness	kurtosis	activity ratio	roughness
8×8	4.17 (0.021)	−241 (2.8×10^−3^)	−3.51 (0.071)	14.4 (0.054)	−541 (0.124)	−5.88 (0.677)
12×12	2.03 (7.2×10^−4^)	−168 (1.1×10^−3^)	−4.62 (0.021)	4.56 (0.042)	−572 (0.052)	−1.71 (0.904)
16×16	1.55 (1.4×10^−4^)	−157 (5.3×10^−4^)	−3.29 (0.087)	2.80 (0.094)	−409 (0.083)	−2.21 (0.879)

Our results show that female faces which are rated the most attractive by men should be the most sparsely coded by the primary visual cortex of these men. The correlation is stronger and more significant with dataset 1 compared with dataset 2. This difference is not explained by sample size (results not shown) but was expected from differences in image processing. Contrary to dataset 1, faces of dataset 2 were (i) presented in colour, which influences rating of face attractiveness [23] and (ii) reveal more hair, which represent many high frequency features influencing more the predicted than the real sparseness in mens’ V1, because our model gives similar weight to any region of an image while people viewing faces typically spend little time scanning hair [24]. In support to this second explanation, *R*^2^ between attractiveness and kurtosis (using 12×12 basis functions) increased from 7 to 11% when hair in dataset 2 was blurred as in dataset 1 before calculating kurtosis (electronic supplementary material, table S2). It is notable that, despite these two limitations, the same trend as in dataset 1 could be detected in dataset 2.

### 3.2 Efficient coding influences attractiveness

Beyond this correlational relationship, what is the effective influence of sparseness on attractiveness? Our results on sparseness were obtained while controlling for three factors: age of women, facial symmetry and skin roughness. In our analyses, the first two factors were never found to be significant (electronic supplementary material, table S1). This is not unexpected given the limited variation in age in our datasets, and the limited associations between attractiveness and symmetry found in previous studies investigating naturally varying asymmetry in faces (for a review, see [25]). Skin roughness was significantly or marginally significant in dataset 1 but not in dataset 2 (table 1). Although we used a classical method of texture analysis based on a grey-level co-occurrence matrix, the method may be limited for modelling texture perception in coloured images [26], thereby explaining the discrepancy between the two datasets. Both facial symmetry and skin roughness are thought to explain a limited fraction of variance in attractiveness [25], and it has been further suggested that their correlations with attractiveness could be driven by third factors [27,28]. Similarly, one cannot exclude that the correlation between sparseness and attractiveness actually reflects the influence of uncontrolled covariates.

Despite the above caveat, and given that we have excluded certain obvious covariates such as skin smoothness, we would nevertheless argue that the magnitude of the correlation between coding sparseness on attractiveness indicates a phenomenon of biological significance. Sparse coding is a ubiquitous strategy, occurring from peripheral sensory systems [29] to higher brain areas [13]. By modelling sparseness in V1 only, it is therefore likely that we underestimate the overall effect of sparse coding on attractiveness.

More importantly, our results are in line with a body of literature suggesting that efficient coding directly determines aesthetics preferences. A century of research in empirical aesthetics has revealed preferences for certain forms and patterns that appear universal, being shared between societies in humans [30] and between species (e.g. [31,32]). Furthermore, these preferences are not domain-specific, being expressed with faces, landscapes as well as simple abstract geometric forms [30]. The best documented of these preferences are for symmetrical, averaged and prototypical forms, curved contours and scale-invariant patterns [30]. As noted by several authors (e.g. [33,34]), these preferred stimuli have in common to be efficiently coded by the perceptual system (in primates, for example, in the retina for curved forms, in the lateral geniculate nucleus for scale-invariant patterns, and in the cognitive areas for prototypical stimuli). In accordance with this efficient coding theory of aesthetics, it has been predicted that stimuli coded sparsely by the perceptual system should be viewed as attractive [34]. To our knowledge, our study is the first to test and support this prediction.

### 3.3 Evolutionary consequences of sparse and efficient coding

We evidenced that faces coded sparsely by the primary visual cortex V1 are more attractive. The primary visual cortex is a generalist brain region that has been shaped through natural selection and development to process the complex statistics of natural scenes [10,35], not to perform a specific task like identifying faces or evaluating their attractiveness. The positive correlation between face attractiveness and sparse coding in our model of V1 simple cells thus indicates that attraction for faces is, at least in part, a perceptual bias driven by the efficient coding strategy of the neuronal circuitry.

The efficient coding bias is expected to influence evolution of communicative traits. Here it is important to clarify what does the attractiveness for a sparse face really mean. From a sparse coding perceptive, the sparsest face would be entirely blank. Yet all the face features have not primarily evolved for communicative purposes. The presence, location and design of the mouth, nostrils, eyebrows and other face features are constrained during development and have been selected to ensure vital physiological functions. In addition, these features are important cues used to evaluate the genetic quality and health state of a potential mate through visual assessments that certainly have primacy over the efficient coding bias. Although, as demonstrated here the efficient coding bias may explain a non-negligible fraction of variance in attractiveness, and we suggest that it may contribute to finely tune the design of sexually selected traits. In other words, the efficient coding bias would not explain why the peacock has a long tail but it could provide explanations for the design of eyespots and other refinements.

The efficient coding bias is probably universal. Sparse coding in particular is used in visual, auditory and olfactory systems of various animals including invertebrates [36,37]. This perceptual bias thus offers a general mechanism for nucleating the association between ornaments and preferences, which could subsequently coevolve, become adaptive or diversify through other mechanisms of sexual selection. Noteworthy, the efficient coding bias could also promote the diversification of signals since the nature of the preferred stimuli can vary between species, populations and even individuals.

Last, the efficient coding bias is tightly linked to the model of sensory exploitation. While this model posits that signals adapt to the external environment to optimize information transmission [38], according to the efficient coding bias, this optimization is further permitted by adaptation of signals to the internal environment of the perceiver. Many studies on sensory exploitation have evidenced that signals are both adapted to the environment and are preferred by the perceiver, but it is largely unknown why the adapted signals are preferred: do they improve signal detection or recognition, the evaluation of information, its reliability? Similarly, it is still unclear why efficiently coded stimuli should be preferred. They could be energetically beneficial to the observer [10,15], but it has been also demonstrated that such stimuli are more precisely coded by the perceptual system and are stored longer in memory [13,15]. Neurophysiologists and behavioural ecologists could both contribute to highlight this question.

Our main result that coding sparseness in observers’ V1 is correlated with facial attractiveness concord with recent advances in psychology and neuroscience, which suggest that aesthetic preferences in part are a perceptual bias favouring efficiently coded stimuli. This implies that the benefits of selecting an aesthetical display can be fortuitous, not adaptive in the context of a specific visual task. These findings support Darwin’s view that mate choice is not necessarily adaptive, but instead is primarily influenced by attraction for pure beauty [1–3].

Modern evolutionary biologists have almost (but see [39]) invariably interpreted preferences for symmetrical, averaged and gender-typical communicative traits under the umbrella of the quality-indicator traits paradigm ([25,28]; see also [32] in birds). All these preferences can also be explained by the efficient coding bias. Future studies should allow unravelling of the relative contribution of the efficient coding bias and of preferences that evolved to assess mate quality in sexual selection.

## Supplementary Material

Table S1. Results of all statistical models. The tables provide, for each explanatory variable of the full model (i.e. without removing non-significant terms), the magnitude of the slope (β) and its standard error (SE), the t-value of the test β=0 and its significance P(t), the residual standard error of the model (RSE), the degrees of freedom (df), and the spearman coefficient of determination (R2) between attractiveness and sparseness. Table S2. Statistical results with hair blured in data set 2. Measure of sparseness = kurtosis, size of receptive fields = 12 ˣ 12 pixels. The tables provide, for each explanatory variable of the full model (i.e. without removing non-significant terms), the magnitude of the slope (β) and its standard error (SE), the t-value of the test β=0 and its significance P(t), the residual standard error of the model (RSE), the degrees of freedom (df), and the spearman coefficient of determination (R2) between attractiveness and kurtosis Figure S1. Example pictures from the two data sets. Data set 1 (right) and data set 2 (left). Figure S2. Measure of facial symmetry and skin roughness. For symmetry (in red), we first positioned 14 points on reliably identified facial features. We drew seven line segments, linking the seven pairs of landmark points P1-P2, P3-P4, P5-P6, P7-P8, P9-P10, P11-P12, and P13-P14. A plane of symmetry (PS) was then identified as a vertical line crossing the mean midpoint of the seven line segments. We measured the absolute difference between the length of P3-P5 and the length of P4-P6, and the absolute distances between segment midpoints and PS. The measure of symmetry is given by the sum of these 8 distances. For skin roughness (in blue), we drew 180ˣ100 pix rectangles with inner-bottom corners located at P13 and P14. We added one 100ˣ180 rectangle on the forehead, centred on PS and bordering (but not including) eyebrows. Entropy was calculated using function entropyfilt in MATLAB, for every distinct 12ˣ12 rectangles embedded within the three rectangles. Skin roughness is given as the average of all entropies.
