# Supplementary material for: Beauty is in the efficient coding of the beholder
Source: R Soc Open Sci. 2016 Mar 2;3(3):160027. doi: 10.1098/rsos.160027 (PMC4821279; doi:10.1098/rsos.160027)
Supplement: Table S1. Results of all statistical models. The tables provide, for each explanatory variable of the full model (i.e. without removing non-significant terms), the magnitude of the slope (β) and its standard error (SE), the t-value of the test β=0 and its significance P(t), the residual standard err [file rsos160027supp1.pdf]

Supplementary Materials

Table S1. Results of all statistical models. The tables provide, for each explanatory variable of the full model (i.e. without removing non-significant terms), the magnitude of the slope (*β*) and its standard error (SE), the *t*-value of the test *β*=0 and its significance *P(t)*, the residual standard error of the model (RSE), the degrees of freedom (df), and the spearman coefficient of determination (R^2) between *attractiveness* and sparseness.

With data set 1, measure of sparseness = *kurtosis*, size of receptive fields = 12×12 pixels:

**Explanatory variable**	***β*(SE)**	***t*-value**	***P(t)***
kurtosis	2.03(0.58)	3.45	7.2×10^{-4}
symmetry	-0.01(0.03)	-0.49	0.624
roughness	-3.51(1.93)	-1.81	0.071
age	-0.17(0.37)	-0.47	0.637
	**RSE(df)**	**R^2**	
	7.64(149)	0.13	

With data set 1, measure of sparseness = *kurtosis*, size of receptive fields = 8×8 pixels:

**Explanatory variable**	***β*(SE)**	***t*-value**	***P(t)***
kurtosis	4.17(1.79)	2.31	0.021
symmetry	0.00(0.01)	-0.14	0.894
roughness	-4.62(1.99)	-2.32	0.021
age	-0.25(0.38)	-0.67	0.503
	**RSE(df)**	**R^2**	
	7.85(149)	0.05	

With data set 1, measure of sparseness = *kurtosis*, size of receptive fields = 16×16 pixels:

**Explanatory variable**	***β*(SE)**	***t*-value**	***P(t)***
kurtosis	1.55(0.39)	3.90	1.4×10^{-4}
symmetry	-0.01(0.03)	-0.53	0.592
roughness	-3.29(1.91)	-1.72	0.087
age	-0.20(0.37)	-0.55	0.581
	**RSE(df)**	**R^2**	
	7.57(149)	0.17	

With data set 1, measure of sparseness = *activity ratio*, size of receptive fields = 12×12 pixels:

**Explanatory variable**	***β*(SE)**	***t*-value**	***P(t)***
activity ratio	-167.9(50.7)	-3.31	1.1×10^{-3}
symmetry	-0.01(0.03)	-0.45	0.613
roughness	-3.12(2.00)	-1.56	0.120
age	-0.24(0.37)	-0.64	0.518
	**RSE(df)**	**R^2**	
	7.66(149)	0.12	

With data set 1, measure of sparseness = *activity ratio*, size of receptive fields = 8×8 pixels:

**Explanatory variable**	***β*(SE)**	***t*-value**	***P(t)***
activity ratio	-240.7(79.2)	-3.03	2.8×10^{-3}
symmetry	-0.01(0.03)	-0.40	0.689
roughness	-3.28(2.02)	-1.62	0.107
age	-0.20(0.37)	-0.55	0.584
	**RSE(df)**	**R^2**	
	7.70(149)	0.09	

With data set 1, measure of sparseness = *activity ratio*, size of receptive fields = 16×16 pixels:

**Explanatory variable**	***β*(SE)**	***t*-value**	***P(t)***
activity ratio	-156.9(44.3)	-3.54	5.3×10^{-4}
symmetry	-0.01(0.03)	-0.50	0.615
roughness	-2.75(2.01)	-1.36	0.173
age	-0.24(0.37)	-0.65	0.517
	**RSE(df)**	**R^2**	
	7.63(149)	0.15	

With data set 2, measure of sparseness = *kurtosis*, size of receptive fields = 12×12 pixels:

**Explanatory variable**	***β*(SE)**	***t*-value**	***P(t)***
kurtosis	4.56(2.20)	2.07	0.042
symmetry	-0.00(0.11)	-0.02	0.983
roughness	-1.71(14.1)	-0.12	0.904
age	-1.87(0.83)	-1.41	0.161
	**RSE(df)**	**R^2**	
	19.99(63)	0.07	

With data set 2, measure of sparseness = *kurtosis*, size of receptive fields = 8×8 pixels:

**Explanatory variable**	***β*(SE)**	***t*-value**	***P(t)***
kurtosis	14.4(8.18)	1.97	0.054
symmetry	-0.01(0.11)	-0.11	0.909
roughness	-5.88(14.0)	-0.24	0.677
age	-1.16(0.85)	-1.37	0.173
	**RSE(df)**	**R^2**	

	20.18(63)	0.07	

With data set 2, measure of sparseness = *kurtosis*, size of receptive fields = 16×16 pixels:

**Explanatory variable**	***β*(SE)**	***t*-value**	***P(t)***
kurtosis	2.8(1.97)	1.69	0.094
symmetry	-0.00(0.11)	-0.00	0.996
roughness	-2.21(14.5)	-0.15	0.879
age	-1.20(0.85)	-1.41	0.163
	**RSE(df)**	**R^2**	
	20.32(63)	0.04	

With data set 2, measure of sparseness = *activity ratio*, size of receptive fields = 12×12 pixels:

**Explanatory variable**	***β*(SE)**	***t*-value**	***P(t)***
activity ratio	-572(290)	-1.98	0.052
symmetry	0.00(0.11)	-0.04	0.969
roughness	-5.88(14.0)	-0.24	0.677
age	-1.16(0.85)	-1.37	0.173
	**RSE(df)**	**R^2**	
	20.23(63)	0.04	

With data set 2, measure of sparseness = *activity ratio*, size of receptive fields = 8×8 pixels:

**Explanatory variable**	***β*(SE)**	***t*-value**	***P(t)***
activity ratio	-541(256)	-1.56	0.124
symmetry	-0.00(0.11)	-0.05	0.959
roughness	-4.62(14.3)	-0.32	0.747
age	-1.18(0.85)	-1.38	0.173
	**RSE(df)**	**R^2**	

	20.41(63)	0.05	

With data set 2, measure of sparseness = *activity ratio*, size of receptive fields = 16×16 pixels:

**Explanatory variable**	***β*(SE)**	***t*-value**	***P(t)***
activity ratio	-409(233)	-1.75	0.083
symmetry	0.00(0.11)	-002	0.984
roughness	-0.89(14.7)	-0.06	0.951
age	-1.16(0.82)	-1.37	0.176

	**RSE(df)**	**R^2**	
	20.33(63)	0.04	

Table S2. Statistical results with hair blured in data set 2. Measure of sparseness = *kurtosis*, size of receptive fields = 12 × 12 pixels. The tables provide, for each explanatory variable of the full model (i.e. without removing non-significant terms), the magnitude of the slope (β) and its standard error (SE), the t-value of the test β=0 and its significance $P(t)$, the residual standard error of the model (RSE), the degrees of freedom (df), and the spearman coefficient of determination (R^2) between *attractiveness* and *kurtosis*.

Explanatory variable	β(SE)	*t*-value	*P(t)*
kurtosis	4.85(2.13)	2.27	0.026
symmetry	0.00(0.11)	0.02	0.985
roughness	-7.25(14.1)	-0.51	0.612
age	-1.39(0.87)	-1.60	0.114
	**RSE(df)**	**R^2**	
	20.46(63)	0.11	

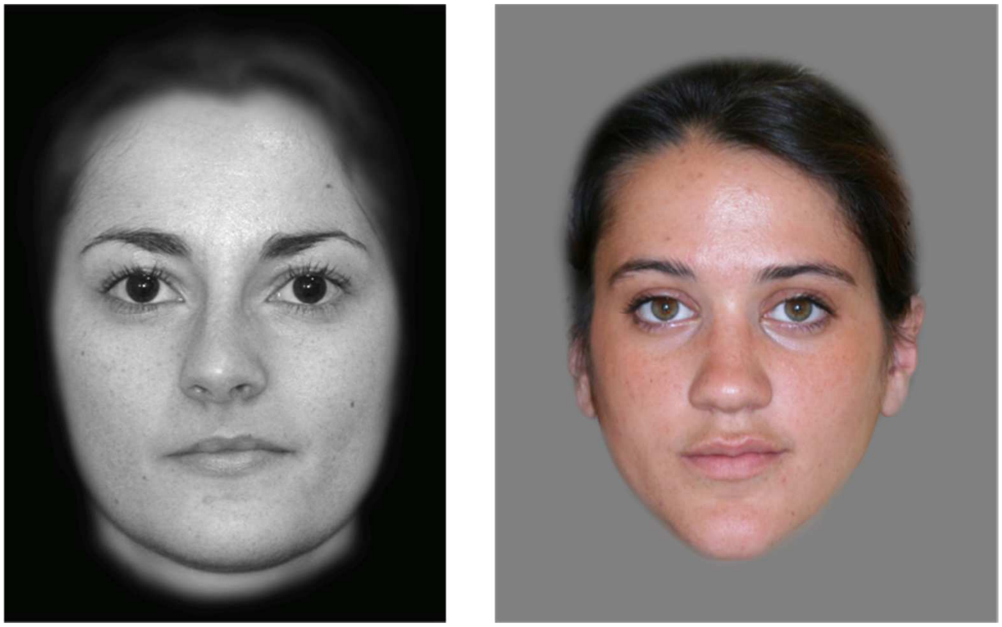

Figure S1. **Example pictures from the two data sets**. Data set 1 (right) and data set 2 (left).

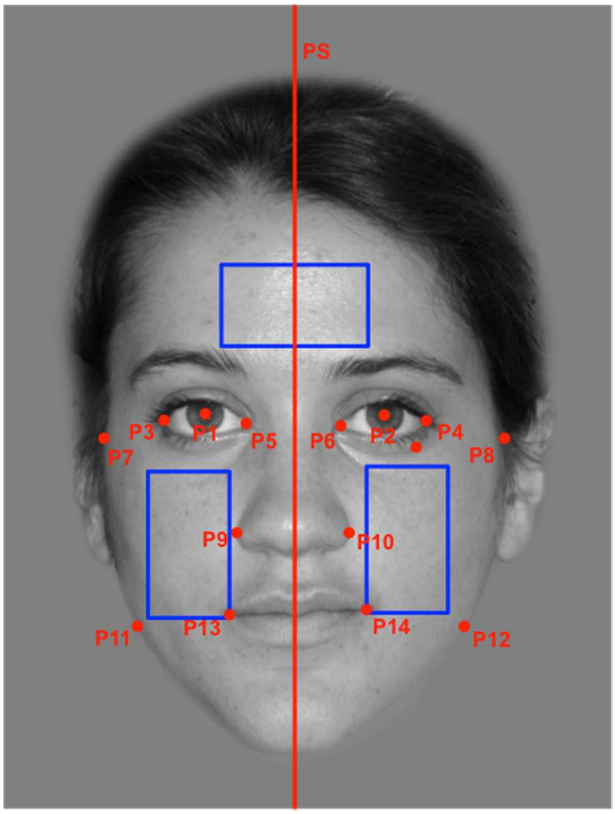

Figure S2. **Measure of facial symmetry and skin roughness**. For symmetry (in red), we first positioned 14 points on reliably identified facial features. We drew seven line segments, linking the seven pairs of landmark points P1-P2, P3-P4, P5-P6, P7-P8, P9-P10, P11-P12, and P13-P14. A plane of symmetry (PS) was then identified as a vertical line crossing the mean midpoint of the seven line segments. We measured the absolute difference between the length of P3-P5 and the length of P4-P6, and the absolute distances between segment midpoints and PS. The measure of symmetry is given by the sum of these 8 distances. For skin roughness (in blue), we drew 180×100 pix rectangles with inner-bottom corners located at P13 and P14. We added one 100×180 rectangle on the forehead, centred on PS and bordering (but not including) eyebrows. Entropy was calculated using function *entropyfilt* in MATLAB, for every distinct 12×12 rectangles embedded within the three rectangles. Skin roughness is given as the average of all entropies.
